# Interleukin-1, tumor necrosis factor-alpha, and transforming growth factor-beta 1 and integrative meniscal repair: influences on meniscal cell proliferation and migration

**DOI:** 10.1186/ar3515

**Published:** 2011-11-16

**Authors:** Katherine M Riera, Nicole E Rothfusz, Rebecca E Wilusz, JB Weinberg, Farshid Guilak, Amy L McNulty

**Affiliations:** 1 Department of Orthopaedic Surgery, Duke University Medical Center, DUMC Box 3093, Durham, NC 27710, USA; 2 Department of Biomedical Engineering, Duke University, DUMC Box 3093, Durham, NC 27710, USA; 3 Department of Medicine, Duke University Medical Center, Durham, NC 27705, USA; 4 Department of Medicine, VA Medical Center, E1006 VA Medical Center, 508 Fulton Street, Durham, NC 27705, USA

## Abstract

**Introduction:**

Interleukin-1 (IL-1) and tumor necrosis factor-α (TNF-α) are up-regulated in injured and osteoarthritic knee joints. IL-1 and TNF-α inhibit integrative meniscal repair; however, the mechanisms by which this inhibition occurs are not fully understood. Transforming growth factor-β1 (TGF-β1) increases meniscal cell proliferation and accumulation, and enhances integrative meniscal repair. An improved understanding of the mechanisms modulating meniscal cell proliferation and migration will help to improve approaches for enhancing intrinsic or tissue-engineered repair of the meniscus. The goal of this study was to examine the hypothesis that IL-1 and TNF-α suppress, while TGF-β1 enhances, cellular proliferation and migration in cell and tissue models of meniscal repair.

**Methods:**

A micro-wound assay was used to assess meniscal cell migration and proliferation in response to the following treatments for 0, 24, or 48 hours: 0 to 10 ng/mL IL-1, TNF-α, or TGF-β1, in the presence or absence of 10% serum. Proliferated and total cells were fluorescently labeled and imaged using confocal laser scanning microscopy and the number of proliferated, migrated, and total cells was determined in the micro-wound and edges of each image. Meniscal cell proliferation was also assessed throughout meniscal repair model explants treated with 0 or 10 ng/mL IL-1, TNF-α, or TGF-β1 for 14 days. At the end of the culture period, biomechanical testing and histological analyses were also performed. Statistical differences were assessed using an ANOVA and Newman-Keuls *post hoc *test.

**Results:**

IL-1 and TNF-α decreased cell proliferation in both cell and tissue models of meniscal repair. In the presence of serum, TGF-β1 increased outer zone cell proliferation in the micro-wound and in the cross section of meniscal repair model explants. Both IL-1 and TNF-α decreased the integrative shear strength of repair and extracellular matrix deposition in the meniscal repair model system, while TGF-β1 had no effect on either measure.

**Conclusions:**

Meniscal cell proliferation *in vivo *may be diminished following joint injury due to the up-regulation of inflammatory cytokines, thereby limiting native cellular repair of meniscal lesions. Therefore, therapies that can promote meniscal cell proliferation have promise to enhance meniscal repair and improve tissue engineering strategies.

## Introduction

The menisci are C-shaped fibrocartilaginous tissues located between the femoral condyles and tibial plateau in the knee. They provide load bearing capabilities, lubrication, proprioception, joint congruity and joint stability for normal biomechanical function of the knee joint [1-4]. Damage to and loss of function of the menisci through sports-related [5] or degenerative tears are associated with pain and degradative changes in the knee joint that ultimately lead to osteoarthritis (OA) [6-11]. Approximately two-thirds of patients with meniscal tears develop radiographic knee OA within 5 to 15 years of injury [12]. Partial excisions and total meniscectomies for the treatment of meniscal tears are strongly associated with articular cartilage loss and the progression of OA [6-11]. Therefore, current orthopaedic practice aims to preserve meniscal integrity and restore function.

The success of clinical repairs depends on a number of factors including age, time to surgery, and the type and location of the meniscal tear. In general, repairs involving the outer one-third of the meniscus, the vascularized "red-red zone", have the highest likelihood of success [13]. Repairs are less favorable in the inner two-thirds of the meniscus, the avascular "white-white zone" [13,14]. However, *in vitro *studies of integrative repair suggest that the intrinsic repair capabilities of the outer and inner zones are similar, supporting the hypothesis that the *in vivo *presence of vasculature aids in the repair of the outer zone [15]. Nonetheless, differences in extracellular matrix and cell composition between the inner and outer zones may also influence repair. The outer zone contains fibroblast-like cells [16,17] that produce predominantly type I collagen [18-20]. The inner zone consists of fibrochondrocyte-like cells [16,17], both type I and II collagen [18,20], and increased aggrecan content relative to the outer zone [14]. Meniscal plugs from the outer zone inserted into inner zone tissue demonstrate enhanced healing, suggesting that repair capability is related to the intrinsic healing potential of the outer region, rather than the vasculature alone [21].

The integrative repair of meniscal lesions is associated with increased cell accumulation in the repair site [22-27]. However, the respective roles of cell proliferation and migration in integrative repair, and the influence of soluble mediators on these processes are not fully understood. An *in vivo *canine model consisting of a fibrin clot surgically inserted into an avascular meniscal defect showed that the clot functioned as a scaffold for cell migration and a chemotactic stimulus for cell proliferation [28]. Furthermore, cells can migrate into an acellular meniscal plug *in vivo *and remodel the tissue [29]. An important factor that may strongly influence meniscal repair is the inflammatory environment within the joint. The inflammatory cytokines interleukin-1 (IL-1) and tumor necrosis factor-alpha (TNF-α) are up-regulated in injured and OA knee joints [30-33]. In addition, IL-1 and TNF-α decrease integrative meniscal repair *in vitro *by increasing matrix metalloproteinase (MMP) activity, sulfated glycosaminoglycan (S-GAG) release, and nitric oxide (NO) production, while simultaneously decreasing cell accumulation and tissue formation at the meniscal repair interface, and ultimately compromising the shear strength of repair [23-27,34]. Initial acute exposure to IL-1 for 1 to 3 days potently suppresses meniscal repair for at least 28 days [27], suggesting that the initial inflammatory environment in a joint post-injury may have long-term degenerative effects. In addition, IL-1 and TNF-α activate other degradative and pro-inflammatory pathways in the meniscus and other joint tissues [30,31,35-37].

While many of the potentially negative effects of IL-1 and TNF-α on meniscal repair have been established at the molecular and tissue levels, the specific effects of these proinflammatory cytokines on meniscal cell migration and proliferation are currently unclear, and several *in vitro *studies have reported conflicting results. In one study, different concentrations of IL-1 caused increased cell migration as compared to controls in bovine meniscal cells isolated from the outer and middle meniscal zones [38]. Conversely, studies with porcine meniscal repair model tissue explants treated with either IL-1 or TNF-α show decreased cell accumulation in the repair interface without a decrease in cell viability, potentially due to a reduction in cell proliferation and/or migration at the repair site [23,25-27].

Anabolic growth factors have been studied as therapeutics to enhance healing of meniscal injuries. The anabolic growth factor transforming growth factor-β1 (TGF-β1) has been shown to increase meniscal cell proliferation in several *in vitro *models, including monolayer, explant culture, and meniscal cells seeded on poly-L-lactide (PLLA) scaffolds and three-dimensional collagen sponges [39-43]. *In vitro *meniscal repair model explants treated with TGF-β1 showed increased cell accumulation in the repair interface and increased integrative repair [24,44]. In the presence of IL-1, TGF-β1 increased the interfacial shear strength of repair compared to IL-1 alone, overcoming some of the potent catabolic effects of IL-1 [24]. Bovine meniscal cells transduced with vectors expressing TGF-β1 and seeded into the avascular inner zone of the meniscus showed increased cellularity and proteoglycan and collagen synthesis [45]. Furthermore, meniscal cells treated with either 10 or 100 ng/mL TGF-β1 showed marked changes in cell morphology, resulting in a phenotype more similar to fibroblast-like cells [39].

The goal of this study was to investigate the effects of the inflammatory cytokines IL-1 and TNF-α, and the growth factor TGF-β1 on proliferation and migration during cell-mediated repair of the meniscus. We hypothesized that IL-1 and TNF-α suppress cellular proliferation and migration of both inner and outer zone meniscal cells, while TGF-β1 enhances cell proliferation and migration of both inner and outer zone cells, in cell and tissue models of meniscal repair. We assessed cell migration and proliferation using a micro-wound assay with isolated inner and outer zone meniscal cells treated with IL-1, TNF-α or TGF-β1. Cells were fluorescently labeled to identify newly proliferated and total cells and were imaged over time to assess the contribution of proliferated and migrated cells to wound healing. Additionally, cell proliferation was assessed in inner and outer zone meniscal repair model explants [15,23,25-27,34] treated with IL-1, TNF-α or TGF-β1 for 14 days. Meniscus healing was investigated by mechanical testing of the repair model explants to determine the interfacial shear strength and histology was performed to visualize tissue repair and cell viability.

## Materials and methods

### Meniscal cell isolation

Medial menisci were aseptically isolated from the knee joints of skeletally mature, two- to three-year-old female pigs obtained from a local abattoir. The menisci were trimmed to remove all ligamentous and synovial tissue and separated into the inner two-thirds and outer one-third zones [14]. Meniscal cells from the inner and outer zones were enzymatically isolated from the tissue by sequential digestion with 1,320 PUK/mL pronase (Calbiochem, San Diego, CA, USA) followed by 0.4% collagenase type I (Worthington, Lakewood, NJ, USA) for three hours, as previously described [46]. After enzymatic isolation, the cells were filtered through a 70 μm filter (BD Biosciences, Bedford, MA, USA) and washed three times in Dulbecco's Modified Eagle's Medium high glucose (DMEM with 4.5 g/L D-glucose; Invitrogen, Carlsbad, CA, USA) containing 1,000 units/mL penicillin/streptomycin and 2.5 μg/mL amphotericin B (Invitrogen). Cells were resuspended at a concentration of 1 × 10^6 ^cells/mL in culture media composed of DMEM, 10% heat inactivated fetal bovine serum (FBS; HyClone, Logan, UT, USA), 0.1 mM non-essential amino acids (Invitrogen), 10 mM 4-(2-hydroxyethyl)-1-piperazineethanesulfonic acid buffer solution (HEPES; Invitrogen), 100 units/mL penicillin/streptomycin, and 37.5 μg/mL L-ascorbic acid 2-phosphate (Sigma-Aldrich, St. Louis, MO, USA). Cells were seeded at a final concentration of 2 × 10^6 ^cells per well in a two-well chambered coverglass slide (Nalge Nunc International, Rochester, NY, USA) that was coated overnight with 50 μg/mL bovine type I collagen (Trevigen, Gaithersburg, MD, USA) in phosphate buffered saline (PBS; Mediatech, Manassas, VA, USA). Cells were incubated for 72 hours at 37°C/5% CO_2_.

### Micro-wounding of meniscal cells

We utilized a micro-wound assay, or scratch test, as described previously [47-49] to assess meniscal cell migration and proliferation in monolayer culture (n = three or more wells per treatment group, each from a different animal). Cells were serum-starved for one hour in serum free culture media (no FBS but contained 2 mg/mL bovine serum albumin (BSA; Invitrogen) [38]). After serum starvation, a single vertical scratch was made in the center of each well with a 200 μL yellow plastic pipette tip (USA Scientific, Ocala, FL, USA) to remove all cells and generate a micro-wound. Immediately, cell debris and media were aspirated and fresh serum free culture media was added containing 10 μM 5-ethylnyl-2'-deoxyuridine (EdU from the Click-iT™ EdU Alexa Fluor® 488 Imaging Kit; Invitrogen), to label DNA in proliferating cells, and the treatments listed in Table 1. Cells were incubated at 37°C/5% CO_2 _for 0, 24, or 48 hours then fixed with 3.8% formaldehyde (VWR International, West Chester, PA, USA), and permeabilized with 0.5% Triton X-100 (Sigma-Aldrich). EdU detection was performed using the manufacturer's protocol for the Click-iT EdU Alexa Fluor 488 Imaging Kit to label proliferated cells. Cells were washed in tris ethylenediaminetetraacetic acid (TE), pH 7.4, stained for 30 minutes in the dark with 1 μM Syto^® ^82 nucleic acid stain (Invitrogen) to label all cells, and washed three times with TE.

**Table 1:** Treatments for micro-wounding experiments

Treatments	Concentrations	Vendor
**Serum**	0, 1%, 5%, 10%	Hyclone (Logan, UT, USA)
**Recombinant porcine IL-1α**	0, 0.1 ng/mL, 1 ng/mL, 10 ng/mL	R & D Systems (Minneapolis, MN, USA)
**Recombinant porcine TNF-α**	0, 0.1 ng/mL, 1 ng/mL, 10 ng/mL	R & D Systems (Minneapolis, MN, USA)
**Porcine TGF-β1**	0, 0.1 ng/mL, 1 ng/mL, 10 ng/mL	R & D Systems(Minneapolis, MN, USA)
**10% Serum and factors**	10% Serum	Hyclone (Logan, UT, USA)
	10% Serum + 10 ng/mL IL-1	see above
	10% Serum + 10 ng/mL TNF-α	see above
	10% Serum + 10 ng/mL TGF-β1	see above

Hyclone (Logan, UT, USA); R & D Systems (Minneapolis, MN, USA)

Cells were visualized and photographed using a laser scanning confocal microscope (LSM 510, Carl Zeiss, Inc., Thornwood, NY, USA). To visualize proliferated cells, an excitation wavelength of 488 nm was used and fluorescence was collected at 505 to 530 nm. Total cells were detected by excitation at 543 nm and fluorescence was collected at >585 nm. In order to visualize a single cell layer, an optical slice of 15 μm was utilized. For each sample, the micro-wound was centered in the field of view and four images at different vertical positions along the scratch were obtained.

### Micro-wounding image analysis

The confocal images were exported as separate green and red channel images from the Zeiss LSM Image Browser software (Carl Zeiss, Inc.). Collected images were analyzed using a custom Matlab (MathWorks, Natick, MA, USA) script. Briefly, each of the four green and red channel images for each sample was thresholded using optimal threshold values that were determined for the green and red channels individually. These images were then converted to a binary image to identify labeled cells. Images were sub-divided into 32 32-pixel regions and the number of cells within each region was counted. Cell counts from all regions were summed across the four images to yield the total number of cells (red) and the total number of proliferating cells (green) for each sample. The total number of migrating cells that did not proliferate was the difference between the two channels. To assess cell migration and proliferation in the micro-wound, cell counts were averaged across the two center strips and to assess cell proliferation at the edge, cell counts from the green channel images were averaged across the four peripheral strips at the far left and right edges of the image. The total cell counts at the edges were also measured on Day 0 images to establish the starting cell density for each meniscal cell population. All data are expressed as a percentage of the starting cell density.

In the micro-wounding assay, all cells that accumulate in the gap have migrated into the wound from the edge. Therefore, all cells that are described as proliferated in the gap have in fact both migrated and proliferated. However, the order in which these cellular activities occurred could not be assessed. Cells that are described as migrated have, therefore, only migrated into the wound and did not proliferate.

### Meniscal repair model system

A previously described meniscal repair model system [15,23-27] was used to assess *in vitro *integrative meniscal repair (n = four or more per treatment group, all from different animals). Cylindrical 5 mm biopsy punches (Miltex, York, PA, USA) were harvested perpendicular to the femoral surface of the meniscus from the inner two-thirds and outer one-third of the tissue. Explants were cut parallel to the meniscal surface with a scalpel to a uniform thickness of 2.5 mm using a custom-made cutting block. To simulate a full-thickness tear, a 3 mm biopsy punch (Miltex) was utilized to make a concentric core in the explant, which was removed and immediately reinserted in the original orientation. Explants were placed in a 24-well plate with DMEM containing 1,000 units/mL penicillin/streptomycin for one hour at 37°C/5% CO_2_. Explants were incubated in the culture media described above for isolated meniscal cells. For cell proliferation experiments, all media included 10 μM EdU. Explants were randomly assigned to one the following treatment groups: control, 10 ng/mL IL-1α, 10 ng/mL TNF-α or 10 ng/mL TGF-β1. Media were changed every 3 days, and explants were cultured for a total of 14 days at 37°C/5% CO_2_.

### Cell proliferation analyses in meniscal repair explants

On Day 14, explants were transected vertically to allow visualization of cells throughout the cross section. Explants were labeled using a modified protocol based on the Click -iT™ EdU Alexa Fluor^® ^488 Imaging Kit. Briefly, explants were fixed with 3.8% formaldehyde for 30 minutes, permeabilized with 0.5% Triton X-100 for 30 minutes, and tagged with the Alexa Fluor dye to label all proliferated cells. To stain all cells, explants were washed in TE, pH 7.4, stained for 30 minutes in the dark with 1 μM Syto^® ^82 nucleic acid stain, and then washed three times with TE.

Cells were visualized and photographed using a confocal laser scanning microscope as described above for the micro-wounding assay. Images were collected more than 50 μm into the face of the sample to ensure that cells damaged during transection were excluded [50]. Two images per location were collected from the outer ring, inner core and repair interface for both the surface and cross section of the explants.

### Meniscal repair model explant image analysis

Data were collected from different areas of the surface and cross-section of the meniscal repair model explants. Measured areas were outlined in Zeiss LSM image browser (Carl Zeiss). The surface interface included a 50 μm region on either side of the interface, inclusive of the interface, at the surface of the tissue. The surface region of the tissue included images from both the inner core and outer ring that were outside this defined interface region. For cross section images, the tissue was divided into three distinct layers based on distance from the surface and cell morphology. The first 50 μm from the surface was defined as the "superficial zone," the next 100 μm was defined as the "middle zone," and the next 300 μm was termed the "deep zone." The cross section interface included a 50 μm region on either side of the interface, inclusive of the interface, for each of these three layers. The cross section of the tissue included images of the cross section from both the inner core and outer ring that were outside the defined interface region.

Confocal images were exported as separate green and red channel images and saved as TIFF files. The images were gray-scaled using Adobe Photoshop and processed using Scion Image (Scion Corp., Frederick, MD, USA) to invert, subtract background by removing 2D streaks, and smooth. Optimal threshold values were determined for the green and red channels individually. Proliferated cells (green) and total cell (red) counts were obtained using intensity thresholds of 75 and 40, respectively, and a minimum particle size of five. Cell counts were obtained for each of the defined regions in the surface and cross sectional planes. Percent cell proliferation was calculated by dividing the number of proliferated cells (green) by the number of total cells (red) in each sample and multiplying by 100 percent.

### Biomechanical testing to assess shear strength of repair

On Day 14, shear strength of repair between the outer ring and inner core of meniscal repair explants was measured with a push-out test [15,23-27] using an Electroforce (ELF) 3200 materials testing system (Bose-EnduraTEC Corporation, Eden Prairie, MN, USA). Briefly, explants were centered in a custom-made apparatus, such that the 3 mm inner core was centered over a 4 mm concentric hole in the bottom of the dish. A 2 mm diameter rod attached to a load cell displaced the inner core at a rate of 0.0833 mm/s until the inner core was dislodged from the outer ring. The force required for displacement was recorded over time. Following the push out test, the inner core was imaged using a digital video camera (Sony Electronics, Park Ridge, NJ, USA) with a 94-mm video lens (Infinity, Boulder, CO, USA) to measure the inner core thickness using LabVIEW Vision Builder AI (National Instruments Corporation, Austin, TX, USA). Shear strength of repair (in kPa) was calculated by dividing the peak force measured during the push out test by the surface area of the interface.

### Histological staining of meniscal explants

On Day 12 of the meniscal repair model explant culture, 0.05% nitroblue tetrazolium chloride (NBT; Invitrogen) was added to the explant culture media for histological analyses. NBT is a cell-permeable compound that is reduced by live cells to form a blue formazan product that remains stable to histological processing and paraffin embedding and has been documented as a live cell marker for chondrocytes [51,52]. At Day 14, explants were fixed overnight in 4% paraformaldehyde (Electron Microscopy Sciences, Hatfield, PA, USA), containing 100 mM sodium cacodylate trihydrate (Electron Microscopy Sciences), pH 7.4 at 4°C. Samples were dehydrated in EtOH, infiltrated with xylene, and paraffin embedded. Sections were stained with 0.02% aqueous fast green (Sigma-Aldrich) to label collagens and Accustain Safranin O solution (Sigma-Aldrich) to identify proteoglycans.

### Statistical analyses

Statistical analyses were performed using Statistica 7.0 (StatSoft Inc., Tulsa, OK, USA). A factorial analysis of variance (ANOVA) and the Newman-Keuls *post hoc *test were performed to determine significant differences (α = 0.05) and the interactive effect of time and treatment in the micro-wounding experiments. In the meniscal repair model explant studies, the interactive effect of treatment and tissue zone (inner and outer) in the surface images and push-out test and of treatment, tissue zone and cross section layer (superficial, middle, and deep) in the cross section images were also determined using a factorial ANOVA and Newman-Keuls *post-hoc *test.

## Results

### The effects of serum on inner and outer zone micro-wound repair

**Figure 1:**
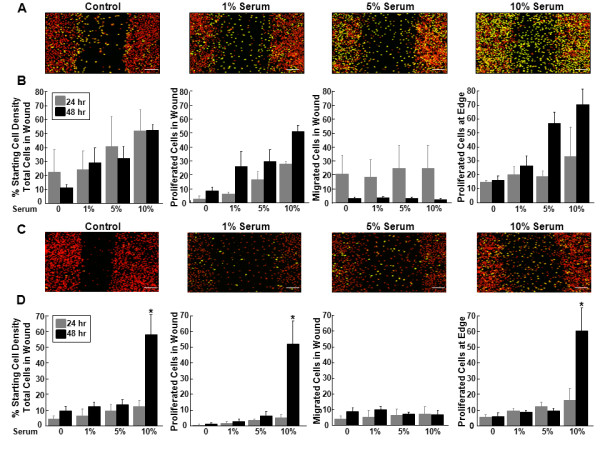
**Serum increased total cells and cell proliferation in inner and outer zone cells**. **(A) **Representative confocal images of the micro-wound from inner zone cells that were treated with 0%, 1%, 5%, or 10% serum for 48 hours after the scratch. In the confocal images, cells that have proliferated are yellow and all cells are labeled red. The scale bar is equal to 100 μm. **(B) **For inner zone cells, total cells in the wound, proliferated cells in the wound, migrated cells in the wound, and proliferated cells at the edge at 24 and 48 hours are graphed as a percentage + standard error of the starting cell density at the edge of the scratch. **(C) **Representative confocal images of the micro-wound from outer zone cells that were treated with 0%, 1%, 5%, or 10% serum for 48 hours after the scratch. **(D) **For outer zone cells, total cells in the wound, proliferated cells in the wound, migrated cells in the wound, and proliferated cells at the edge at 24 and 48 hours are graphed as a percentage + standard error of the starting cell density at the edge of the scratch.*: *P *< 0.01 compared to all other treatments.

Serum treatment of meniscal cells from both the inner (Figure 1A) and outer zones (Figure 1C) resulted in increased accumulation of proliferated cells in the micro-wound. For inner zone meniscal cells, 10% serum increased the total number of cells in the wound as compared to the control (Figure 1B, P < 0.05), increased the percentage of proliferated cells in the wound compared to all other treatments (*P *< 0.005), and enhanced cellular proliferation away from the wound over the control and 1% serum treatments (*P *< 0.05). Additionally, 5% serum promoted cellular proliferation in the wound over the control treatment (*P *< 0.05). There was also an effect of time in the inner zone cells, with increased proliferation at both the edge and in the wound at 48 hours (*P *< 0.005), while the number of cells that had migrated but not proliferated in the wound decreased from 24 to 48 hours (*P *< 0.05).

In outer zone meniscal cells, 10% serum increased the total number of cells in the wound (Figure 1D, P < 0.005) and the percentage of proliferated cells in both the wound (*P *< 0.01) and at the edge (*P *< 0.005), as compared to all other treatments. On average, treatment with 10% serum for 48 hours resulted in a six-fold increase in the total number of cells in the wound and in the proliferated cells at both the edge and in the wound (*P *< 0.01). In addition, there was an effect of time in the outer zone cells, with the total number of cells (*P *< 0.005) and the proliferated cells in the wound being greatest at 48 hours (*P *< 0.01). No differences were detected in cells that migrated but did not proliferate in the wound of outer zone cells.

### The effects of IL-1 on inner and outer zone micro-wound repair

**Figure 2:**
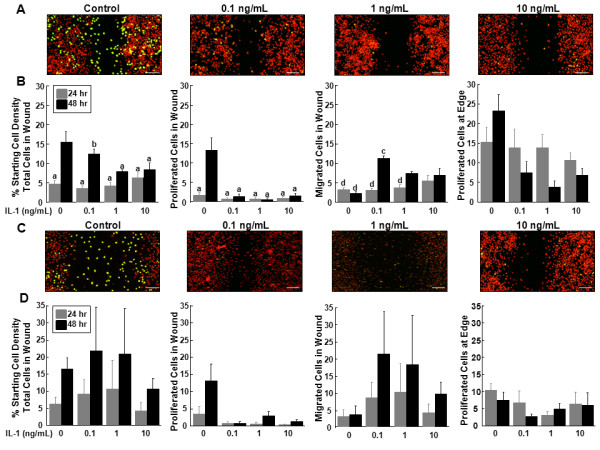
**IL-1 decreased cell proliferation in the wound**. **(A) **Representative confocal images of the micro-wound from inner zone cells that were treated with 0, 0.1 ng/mL, 1 ng/mL, or 10 ng/mL IL-1 for 48 hours after the scratch. In the confocal images, cells that have proliferated are yellow and all cells are labeled red. The scale bar is equal to 100 μm. **(B) **For inner zone cells, total cells in the wound, proliferated cells in the wound, migrated cells in the wound, and proliferated cells at the edge at 24 and 48 hours are graphed as a percentage + standard error of the starting cell density at the edge of the scratch. a: *P *< 0.01 compared to control at 48 hours; b: *P *< 0.05 compared to all other treatments at 24 hours; c: *P *< 0.005 compared to all other treatments; d: *P *< 0.05 compared to 1 ng/mL and 10 ng/mL at 48 hours. **(C) **Representative confocal images of the micro-wound from outer zone cells that were treated with 0, 0.1 ng/mL, 1 ng/mL, or 10 ng/mL IL-1 for 48 hours after the scratch. **(D) **For outer zone cells, total cells in the wound, proliferated cells in the wound, migrated cells in the wound, and proliferated cells at the edge at 24 and 48 hours are graphed as a percentage + standard error of the starting cell density at the edge of the scratch.

IL-1 treatment of meniscal cells from the inner (Figure 2A) or outer zones (Figure 2C) resulted in decreased accumulation of proliferated cells in the micro-wound. As compared to the inner zone control at 48 hours, the overall total number of cells in the wound and the proliferated cells in the wound were significantly decreased by IL-1 treatment (Figure 2B, P < 0.01). However, 0.1 ng/mL IL-1 at 48 hours showed an increase in the total cells in the wound, as compared to all other treatments at 24 hours (*P *< 0.05), and a corresponding increase in the number of cells that migrated but did not proliferate in the wound, as compared to all other treatments at both 24 and 48 hours (*P *< 0.005). There was a significant increase in the number of migrated cells in the wound at 48 hours in the 1 ng/mL and 10 ng/mL IL-1 treatment groups (*P *< 0.05). Overall for inner zone cells, the control treatment caused the greatest proliferation at the edge (*P *< 0.05) and in the wound (*P *< 0.0005) and decreased the number of cells that migrated but did not proliferate in the wound (*P *< 0.005). There was also an effect of time, with 48 hours showing increased total cells, proliferated cells and migrated cells in the wound, as compared to the 24-hour time point (*P *< 0.001).

In the outer zone meniscal cells, IL-1 treatment caused a significant decrease in the number of proliferated cells in the wound, as compared to control (Figure 2D, P < 0.05). However, IL-1 did not have a significant effect on the total cell numbers in the wound, migrated cells in the wound, or the proliferated cells at the edge in the outer zone meniscal cells.

### The effects of TNF-α on inner and outer zone micro-wound repair

**Figure 3:**
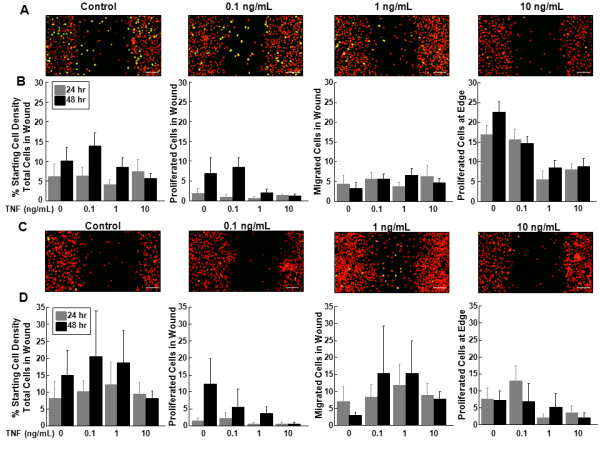
**TNF-α treatment decreased inner zone cell proliferation at the edge**. **(A) **Representative confocal images of the micro-wound from inner zone cells that were treated with 0, 0.1 ng/mL, 1 ng/mL, or 10 ng/mL TNF-α for 48 hours after the scratch. In the confocal images, cells that have proliferated are yellow and all cells are labeled red. The scale bar is equal to 100 μm. **(B) **For inner zone cells, total cells in the wound, proliferated cells in the wound, migrated cells in the wound, and proliferated cells at the edge at 24 and 48 hours are graphed as a percentage + standard error of the starting cell density at the edge of the scratch. **(C) **Representative confocal images of the micro-wound from outer zone cells that were treated with 0, 0.1 ng/mL, 1 ng/mL, or 10 ng/mL TNF-α for 48 hours after the scratch. **(D) **For outer zone cells, total cells in the wound, proliferated cells in the wound, migrated cells in the wound, and proliferated cells at the edge at 24 and 48 hours are graphed as a percentage + standard error of the starting cell density at the edge of the scratch.

Meniscal cells from the inner (Figure 3A), but not the outer zone (Figure 3C), showed diminished accumulation of proliferated cells in the micro-wound with increasing concentrations of TNF-α. In the inner zone cells, proliferation at the edge was diminished by all concentrations of TNF-α (Figure 3B, P < 0.05), as compared to control. In addition, the 1 and 10 ng/mL concentrations of TNF-α caused significant decreases in proliferation at the edge, as compared to 0.1 ng/mL TNF-α (*P *< 0.005). At 48 hours, proliferation in the wound was significantly higher than at 24 hours (*P *< 0.05). In the inner zone cells treated with TNF-α, there were no differences in the total cells in the wound or migrated cells in the wound.

In the outer zone cells, there was a trend towards decreased proliferation at the edge (Figure 3D, P = 0.17) and in the wound (*P *= 0.46) with TNF-α treatment but these changes were not significant. TNF-α treatment did not alter the total number of cells in the wound or the number of cells that had migrated but not proliferated in the wound.

### The effects of TGF-β1 on inner and outer zone micro-wound repair

**Figure 4:**
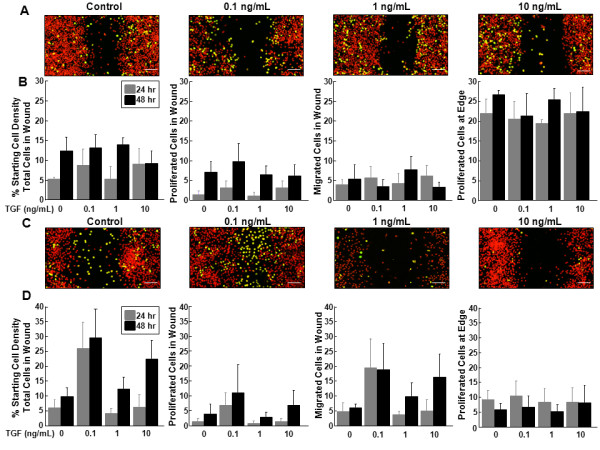
**Low concentrations of TGF-β1 increased total outer zone cells in the wound**. **(A) **Representative confocal images of the micro-wound from inner zone cells that were treated with 0, 0.1 ng/mL, 1 ng/mL, or 10 ng/mL TGF-β1 for 48 hours after the scratch. In the confocal images, cells that have proliferated are yellow and all cells are labeled red. The scale bar is equal to 100 μm. **(B) **For inner zone cells, total cells in the wound, proliferated cells in the wound, migrated cells in the wound, and proliferated cells at the edge at 24 and 48 hours are graphed as a percentage + standard error of the starting cell density at the edge of the scratch. **(C) **Representative confocal images of the micro-wound from outer zone cells that were treated with 0, 0.1 ng/mL, 1 ng/mL, or 10 ng/mL TGF-β1 for 48 hours after the scratch. **(D) **For outer zone cells, total cells in the wound, proliferated cells in the wound, migrated cells in the wound, and proliferated cells at the edge at 24 and 48 hours are graphed as a percentage + standard error of the starting cell density at the edge of the scratch.

In the inner zone meniscal cells, there were no observable changes in cell accumulation or proliferation (Figure 4A). For the inner zone cells, total cells (Figure 4B, P < 0.05) and proliferated cells in the wound (*P *< 0.005) increased with time. No changes were observed with TGF-β1 treatment in the cells that proliferated at the edge or in cells that migrated but did not proliferate in the wound.

On the other hand, in the outer zone cells 0.1 ng/mL TGF-β1 increased cell accumulation (Figure 4C). This concentration of TGF-β1 also significantly increased the total cell number in the wound of the outer zone cells, as compared to the control and 1 ng/mL TGF-β1 treatment groups (Figure 4D, P < 0.05). However, TGF-β1 treatment of outer zone cells did not alter the percentage of proliferated cells in the wound, proliferated cells at the edge, or the number of cells that had migrated into the wound but not proliferated.

### The effects of IL-1, TNF-α, and TGF-β1 in the presence of serum on inner and outer zone micro-wound repair

**Figure 5:**
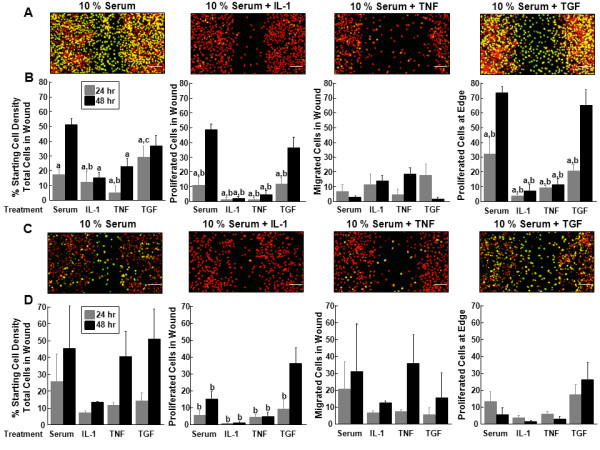
**In the presence of serum, IL-1 and TNF-α decreased, while TGF-β1 increased, cell proliferation**. **(A) **Representative confocal images of the micro-wound from inner zone cells that were treated with 10% serum, 10% serum + 10 ng/mL IL-1, 10% serum + 10 ng/mL TNF-α, or 10% serum + 10 ng/mL TGF-β1 for 48 hours after the scratch. In the confocal images, cells that have proliferated are yellow and all cells are labeled red. The scale bar is equal to 100 μm. **(B) **For inner zone cells, total cells in the wound, proliferated cells in the wound, migrated cells in the wound, and proliferated cells at the edge at 24 and 48 hours are graphed as a percentage + standard error of the starting cell density at the edge of the scratch. a: *P *< 0.05 compared to 10% serum at 48 hour; b: *P *< 0.05 compared to serum + TGF-β1 at 48 hours; c: *P *< 0.05 compared to serum + TNF-α at 24 hours. **(C) **Representative confocal images of the micro-wound from outer zone cells that were treated with 10% serum, 10% serum + 10 ng/mL IL-1, 10% serum + 10 ng/mL TNF-α, or 10% serum + 10 ng/mL TGF-β1 for 48 hours after the scratch. **(D) **For outer zone cells, total cells in the wound, proliferated cells in the wound, migrated cells in the wound, and proliferated cells at the edge at 24 and 48 hours are graphed as a percentage + standard error of the starting cell density at the edge of the scratch.

In the presence of serum, IL-1 and TNF-α treatment of meniscal cells from both the inner (Figure 5A) and outer zones (Figure 5C) resulted in decreased accumulation of proliferated cells in the micro-wound. For inner zone cells, both IL-1 and TNF-α decreased total cell numbers in the wound and the percentage of proliferated cells in the wound and at the edge, as compared to 10% serum treatment for 48 hours (Figure 5B, P < 0.05). In addition, IL-1 and TNF-α suppressed cell proliferation in the wound and at the edge compared to TGF-β1 treatment for 48 hours (*P *< 0.05). Even at 24 hours, TNF-α suppressed total cells in the wound relative to TGF-β1 treatment (*P *< 0.05). There was an increase in the total number of inner zone cells in the wound and proliferated cells in the wound and at the edge over time (*P *< 0.05). None of the tested factors affected inner zone cell migration into the wound in the presence of serum.

In the outer zone cells, TGF-β1 treatment at 48 hours significantly increased cell proliferation in the wound compared to all other treatments (Figure 5D, P < 0.05). In addition, TGF-β1 treatment also promoted cell proliferation at the edge (*P *< 0.05). Furthermore, total cells (*P *< 0.05) and proliferated cells in the wound (*P *< 0.005) increased over time. There was no effect on cell migration into the wound of outer zone cells with the different factors in the presence of serum.

### The effects of IL-1 on cellular proliferation in meniscal repair model explants

**Figure 6:**
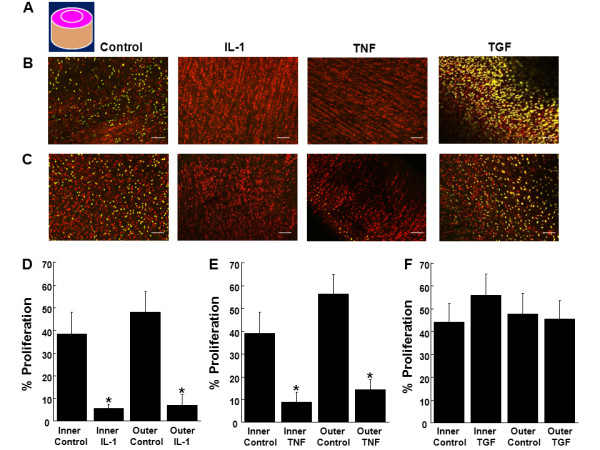
**IL-1 and TNF-α decreased cell proliferation at the tissue surface**. **(A) **Schematic of a meniscal repair explant, indicating the surface of the tissue that was analyzed in pink. Representative confocal images of the surface of **(B) **inner or **(C) **outer zone meniscal repair explants that were treated with 0, 10 ng/mL IL-1, 10 ng/mL TNF-α, or 10 ng/mL TGF-β1 for 14 days. In the confocal images, cells that have proliferated are yellow and all cells are labeled red. The scale bar is equal to 100 μm. Inner and outer zone explants were treated with control media and **(D) **10 ng/mL IL-1, **(E) **10 ng/mL TNF-α, or **(F) **10 ng/mL TGF-β1 for 14 days. The data are graphed as a percentage of proliferated cells at the surface of the tissue + standard error. *: *P *< 0.00005 compared to control.

**Figure 7:**
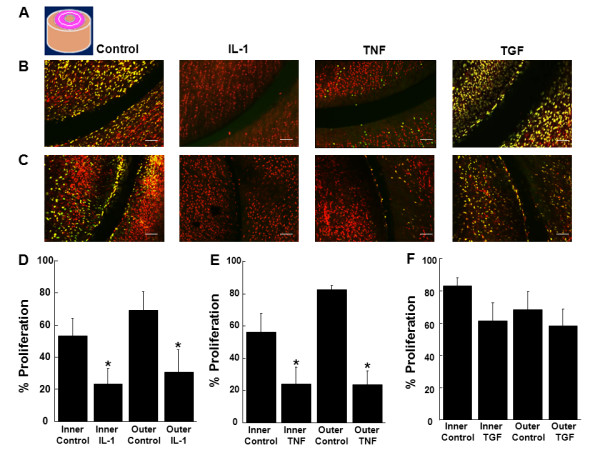
**IL-1 and TNF-α decreased cell proliferation at the surface interface of the meniscal repair explants**. **(A) **Schematic of a meniscal repair explant, indicating the surface interface of the tissue that was analyzed in pink, which included the interface and 50 μm of tissue on either side of the interface. Representative confocal images of the surface interface of **(B) **inner or **(C) **outer zone meniscal repair explants that were treated with 0, 10 ng/mL IL-1, 10 ng/mL TNF-α, or 10 ng/mL TGF-β1 for 14 days. In the confocal images, cells that have proliferated are yellow and all cells are labeled red. The scale bar is equal to 100 μm. Inner and outer zone explants were treated with control media and **(D) **10 ng/mL IL-1, **(E) **10 ng/mL TNF-α, or **(F) **10 ng/mL TGF-β1 for 14 days. The data are graphed as a percentage of proliferated cells at the surface interface of the tissue + standard error. *: *P *< 0.005 compared to control.

**Figure 8:**
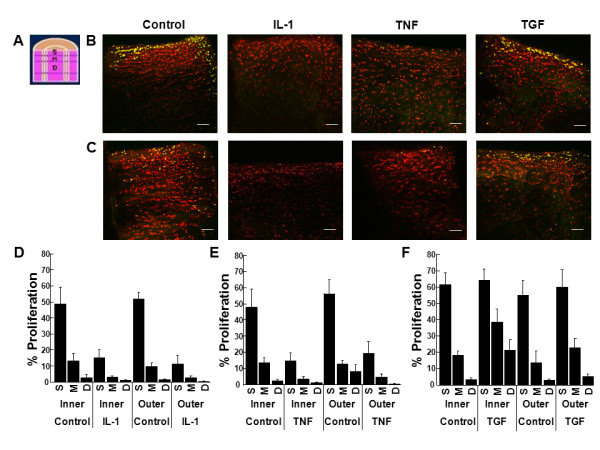
**IL-1 and TNF-α decreased cell proliferation and TGF-β1 increased cell proliferation throughout the cross section**. **(A) **Schematic of a meniscal repair explant, indicating the cross-section of the tissue that was analyzed in pink. The cross-section was divided into three layers: S = superficial layer that comprised the first 50 μm of the tissue, M = middle layer that contained the next 100 μm of tissue, and D = deep layer that consisted of the next 300 μm of meniscal tissue. Representative confocal images of the cross-section of **(B) **inner or **(C) **outer zone meniscal repair explants that were treated with 0, 10 ng/mL IL-1, 10 ng/mL TNF-α, or 10 ng/mL TGF-β1 for 14 days. In the confocal images, cells that have proliferated are yellow and all cells are labeled red. The scale bar is equal to 100 μm. Inner and outer zone explants were treated with control media and **(D) **10 ng/mL IL-1, **(E) **10 ng/mL TNF-α, or **(F) **10 ng/mL TGF-β1 for 14 days. The data are graphed as a percentage of proliferated cells in the cross-section superficial (S), middle (M), and deep (D) layers of the tissue + standard error.

**Figure 9:**
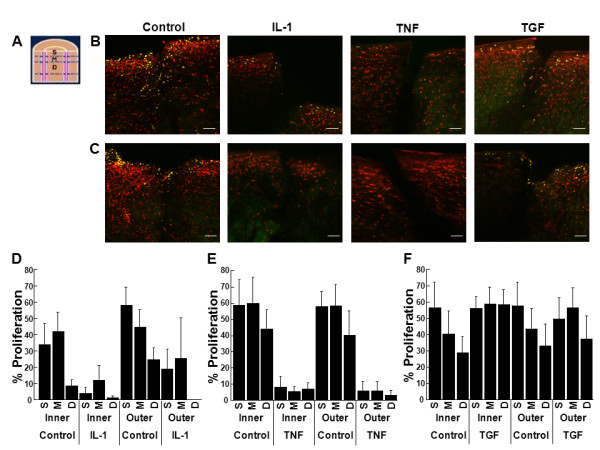
**IL-1 and TNF-α decreased cell proliferation throughout the cross section repair interface**. **(A) **Schematic of a meniscal repair explant, indicating the cross-section interface of the tissue that was analyzed in pink, which included the interface and 50 μm of tissue on either side of the interface. The cross-section interface was divided into three layers: S = superficial layer that comprised the first 50 μm of the tissue, M = middle layer that contained the next 100 μm of tissue, and D = deep layer that consisted of the next 300 μm of meniscal tissue. Representative confocal images of the cross-section interface of **(B) **inner or **(C) **outer zone meniscal repair explants that were treated with 0, 10 ng/mL IL-1, 10 ng/mL TNF-α, or 10 ng/mL TGF-β1 for 14 days. In the confocal images, cells that have proliferated are yellow and all cells are labeled red. The scale bar is equal to 100 μm. Inner and outer zone explants were treated with control media and **(D) **10 ng/mL IL-1, **(E) **10 ng/mL TNF-α, or **(F) **10 ng/mL TGF-β1 for 14 days. The data are graphed as a percentage of proliferated cells in the cross-section repair interface superficial (S), middle (M), and deep (D) layers of the tissue + standard error.

Cellular proliferation at the meniscal tissue surface (Figure 6B, C), surface interface (Figure 7B, C), cross-section (Figure 8B, C), and cross-section interface (Figure 9B, C) were decreased by IL-1 in both inner and outer meniscal repair explants. In both the inner and outer zone explants, IL-1 potently inhibited cell proliferation at the tissue surface (Figure 6D, P < 0.00005) and the surface interface (Figure 7D, P < 0.005). In addition, IL-1 decreased cell proliferation throughout the cross-section (Figure 8D, P < 0.000005) and cross-section interface (Figure 9D, P < 0.0001). In the cross-section, there was a significant difference between all layers with the superficial layer having the highest percentage of proliferated cells and the deep layer having the lowest percentage (Figure 8D, P < 0.05). Furthermore, there was an interactive effect of IL-1 and cross-section layer (*P *< 0.000005). In the cross-section interface, the deep layer had significantly less proliferation than the superficial and middle layers of the tissue (Figure 9D, P < 0.01). Overall in the cross-section interface, cellular proliferation was higher in the outer zone meniscal repair model explants, as compared to the explants from the inner zone (*P *< 0.05).

### The effects of TNF-α on cellular proliferation in meniscal repair model explants

Cellular proliferation at the meniscal tissue surface (Figure 6B, C), surface interface (Figure 7B, C), cross-section (Figure 8B, C), and cross-section interface (Figure 9B, C) were decreased in the presence of TNF-α in both inner and outer meniscal repair explants. TNF-α strongly inhibited cell proliferation at the tissue surface (Figure 6E, P < 0.00005) and the surface interface (Figure 7E, P < 0.005) in explants from both zones. Furthermore, TNF-α reduced cell proliferation throughout the cross-section (Figure 8E, P < 0.000005) and cross-section interface (Figure 9E, P < 0.000005). There was also a significant effect of cross-section layer with the superficial layer containing significantly more proliferated cells than the middle and deep layers (Figure 8E, P < 0.0005). In addition, there was an interactive effect of TNF-α and cross-section layer (*P *< 0.00005).

### The effects of TGF-β1 on cellular proliferation in meniscal repair model explants

In both the inner and outer meniscal repair explants, TGF-β1 treatment did not appear to alter cellular proliferation at the meniscal tissue surface (Figure 6B, C), surface interface (Figure 7B, C), cross-section (Figure 8B, C), or cross-section interface (Figure 9B, C). In both inner and outer zone explants, TGF-β1 had no effect on cellular proliferation in meniscal repair model explants at the tissue surface (Figure 6F), the surface interface (Figure 7F), or the cross-section interface (Figure 9F). However, overall TGF-β1 increased cellular proliferation in the tissue cross-section (Figure 8F, P < 0.05). While there was a significant decrease in cellular proliferation throughout the depth of the meniscus cross-section (Figure 8F, P < 0.005), TGF-β1 most noticeably up-regulated proliferation in the middle and deep layers.

### The effects of IL-1, TNF-α, and TGF-β1 on the shear strength of integrative repair

**Figure 10:**
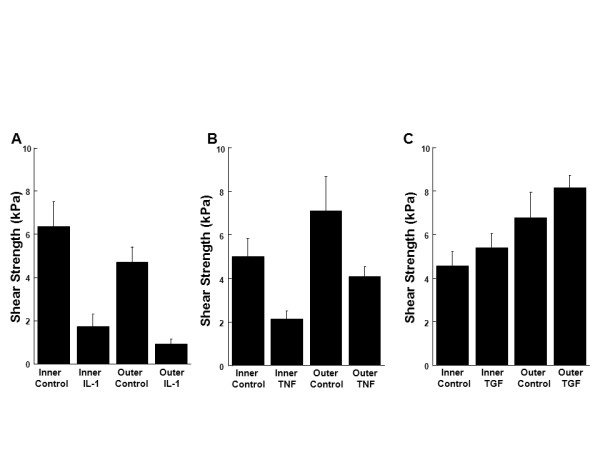
**IL-1 and TNF-α decreased the integrative shear strength of repair**. Inner and outer zone explants were treated with control media and **(A) **10 ng/mL IL-1, **(B) **10 ng/mL TNF-α, or **(C) **10 ng/mL TGF-β1 for 14 days. The data are graphed as shear strength of repair in kPa + standard error.

In the inner and outer zone meniscal explants, both IL-1 (Figure 10A, P < 0.0005) and TNF-α (Figure 10B, P < 0.005) significantly decreased the integrative shear strength of repair. TGF-β1 (Figure 10C) had no effect on the shear strength of the meniscal repair model explants. Outer zone meniscal repair model explants demonstrated increased shear strength of repair, as compared to inner zone explants, when treated with TNF-α (Figure 10B, P < 0.05) and TGF-β1 (Figure 10C, P < 0.005).

### The effects of IL-1, TNF-α and TGF-β1 on tissue repair and cell viability

**Figure 11:**
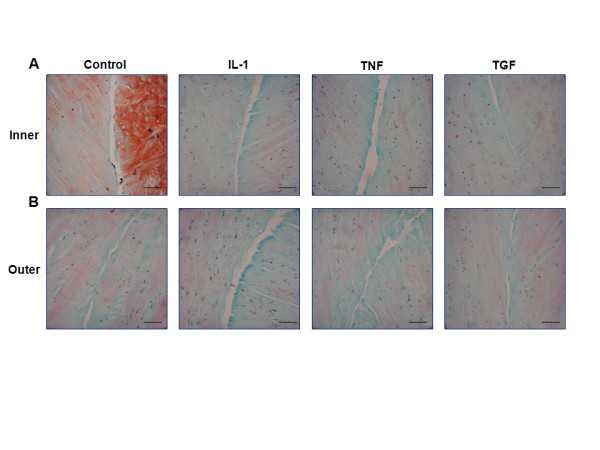
**IL-1 and TNF-α decreased tissue repair but did not decrease cell viability**. Representative histology images of the **(A) **inner or **(B) **outer zone meniscal repair explants that were treated with 0, 10 ng/mL IL-1, 10 ng/mL TNF-α, or 10 ng/mL TGF-β1 for 14 days. In the histology images, collagen fibers are stained green, proteoglycans are stained red, and viable cells are stained dark blue. The scale bar is equal to 100 μm.

Histological analysis revealed healing of the meniscal defect in both inner and outer repair model explants under control conditions (Figures 11A, B). Control inner zone explants stained strongly with safranin O, indicating a relative abundance of proteoglycans, as compared to outer zone samples. In both inner and outer zone explants from the control and TGF-β1 treated groups, the repair interface was filled with an extracellular matrix that stained strongly with fast green, indicating the presence of collagen fibers. No visible tissue repair was detected in explants that were treated with either IL-1 or TNF-α. Cell viability, as indicated by NBT staining, was not altered in any of the treatment groups.

## Discussion

Our results demonstrate that the proinflammatory cytokines IL-1 and TNF-α decreased cell proliferation in both cell and tissue models of meniscal repair. In the presence of serum, the anabolic growth factor TGF-β1 increased outer zone cell proliferation in the micro-wound and in the cross section of meniscal repair model explants. Furthermore, both IL-1 and TNF-α decreased the integrative shear strength of repair and extracellular matrix deposition in the meniscal repair model system, while TGF-β1 had no effect on either measure. Therefore, our results support our hypothesis that the inhibition of cell accumulation and integrative repair by IL-1 and TNF-α is likely due to suppression of cellular proliferation but not migration of cells into meniscal micro-wounds. These results suggest that *in vivo*, meniscal cell proliferation may be diminished following joint injury due to the up-regulation of inflammatory cytokines, thereby limiting native cellular repair of meniscal lesions. Therefore, therapies that can promote meniscal cell proliferation have promise to enhance meniscal repair and improve tissue engineering strategies.

Serum has been shown to promote proliferation in many cell types, including chondrocytes [53,54]. Likely growth factors present in the serum promoted healing of the micro-wound [55]. However, inner and outer zone cells exhibited distinct responses in the micro-wound assay. The inner zone cells showed increased cell proliferation in response to 5% and 10% serum, while outer zone cells were only stimulated by 10% serum. The inner zone cells may be more sensitive to serum stimulation due to the lack of prior exposure to the contents of the vasculature in the context of the meniscus [14]. In addition, for inner zone cells, the percentage of cells that migrated but did not proliferate decreased over time, suggesting that the cells are migrating into the wound and then proliferating to repair the defect.

IL-1 treatment suppressed cell proliferation but increased migration in inner zone cells at the wound, although the enhanced migration was insufficient to overcome the suppression of proliferation in order to repair the micro-wound. On the other hand, IL-1 treatment of outer zone cells decreased proliferation but did not alter cell migration into the micro-wound. In other studies, outer and middle zone meniscal cells that grew out of bovine menisci over two to three weeks showed increased chemotaxis in response to four hours of 1 to 100 ng/mL IL-1 [38]. In this study, we did not assess the chemotactic response of the porcine meniscal cells but the differences in our results may be due to the differences in exposure time to IL-1. In the presence of serum, IL-1 treatment of inner zone cells suppressed total cell accumulation and proliferation but had no effect on migration. These experimental conditions are most similar to our explant growth conditions, and the results of these experiments are consistent. In porcine articular chondrocytes, F-actin content is increased after 1 hour of 10 ng/mL IL-1, showing punctate staining at the periphery but this effect is not observed after 12 hours of IL-1 treatment [56]. In tenocytes treated with 100 pM IL-1β for five days, proliferation rate was unchanged; however, actin filaments were disrupted while microtubule structure was unchanged [57]. In addition, chondrocytes treated with exogenous NO, a downstream mediator of IL-1 signaling, showed inhibition of chondrocyte migration and disruption of actin filament assembly [54]. Therefore, disruption of the actin cytoskeleton may be contributing to the IL-1 mediated suppression of proliferation observed in our injury models.

The effect of TNF-α in suppression of proliferation was not as robust as that observed with IL-1, consistent with our previous observations of the different potencies of equal concentrations of IL-1 and TNF-α on meniscal repair [25]. In addition, TNF-α had no effect on the migration of meniscal cells after micro-wounding. TNF-α treatment of human umbilical vein endothelial cells (HUVECs) caused microtubule bundling [58]; perhaps this reorganization prevents cellular proliferation in response to TNF-α. Furthermore, in the presence of serum, TNF-α treatment of inner zone cells suppressed total cell accumulation and proliferation but had no effect on migration, consistent with our explant experiments.

Similar to our results with TGF-β1 treatment, in other studies using isolated rabbit meniscal cells cultured in 10% FBS and equivalent concentrations of TGF-β1, there was no effect of TGFβ1on cell proliferation at 48 hours [59]. TGF-β1 has been shown to increase F-actin levels in isolated chondrocytes [60] and increase actin extensions and lamellar ruffling in agarose embedded chondrocytes [61]. In other studies, 3T3 fibroblasts treated with TGF-β1 did not migrate or proliferate and contained stabilized microtubules [62], consistent with the overall effects observed in this study.

In the micro-wounding experiments, overall the responses of the cells at the site of the injury and away from the wound were similar for the different treatments. These data suggest that the effect of the cytokines were stronger than any local factors that may be released in response to the wound. However, IL-1 treatment of outer zone cells and TNF-α treatment of inner cells resulted in differential responses between the cells at the site of the wound and at the edge. In these conditions, local factors produced by the wounded cells [63] may have altered the global response to the cytokine treatment [64], resulting in different responses of the cells at the site of the injury and those away from the wound.

The cellular proliferation measured in meniscal repair model explants in this study is consistent with results from previous studies. In particular, using fresh or frozen meniscal plugs in avascular sheep meniscal injuries treated with 50 ng/mL TGF-β1 for eight weeks also demonstrated no difference in cell density or proliferation but cells further from the tissue surface proliferated in response to TGF-β1 [65]. In addition, our data are consistent with an *in vivo *canine model in which superficial layer cells appeared to be the most active in wound repair of meniscal tissue plugs [29]. Taken together, these results suggest that the superficial cells of the meniscus may be integral in initiating and modulating the repair response.

The decreased cellular proliferation by IL-1 and TNF-α correlates with the decreased integrative shear strength of repair. In addition, these data are consistent with our previous studies that have shown that IL-1 and TNF-α suppressed integrative meniscal repair and decreased cell accumulation in the repair interface [23,25]. Additionally, the general lack of an effect on cell proliferation in response to TGF-β1 treatment is consistent with the mechanical testing data. Previously, we have shown that 1 ng/mL TGF-β1 promoted integrative repair but 10 ng/mL TGF-β1 did not [24]. In adult bovine meniscal repair explants, 10 ng/mL TGF-β3 increased the shear strength of repair at eight weeks but not four weeks [44], suggesting that longer times in culture may be necessary to see the beneficial effects of TGF-β1 on meniscal repair. Scaffolds containing TGF-β3 increased chemotaxis of cells and articular cartilage regeneration in a rabbit model, as compared to scaffolds without TGF-β3 [66], suggesting differential responses of cells to the different isoforms of TGF-β. Interestingly, the outer zone explants showed increased shear strength of repair in the TNF-α and TGF-β1 treatment groups, as compared to inner zone explants. This result is similar to the two-week time point in a previous study, but these differences disappeared over extended culture periods [15].

Cell viability was not altered by any of the treatments in this study, suggesting that the decreased repair in the presence of IL-1 and TNF-α was not due to induction of cell death by these cytokines. The inner zone control samples stained more strongly for proteoglycans than the outer zone samples, reflecting the intrinsic composition of the meniscal tissue [14]. Histological staining revealed the presence of a predominantly collagen-rich matrix bridging the interface in control and TGF-β1 treated samples, whereas reparative tissue was largely absent in IL-1 and TNF-α treated explants. New protein synthesis, in particular collagen deposition [67] and cross-linking [68,69], are required for successful integrative repair in cartilage repair model systems.

While isolated inner and outer zone cells demonstrated different responses to the various treatments, cells of the inner and outer zone meniscal repair model explants exhibited similar responses. Recently, isolated outer zone cells have also been shown to migrate faster and have lower adhesion strength than inner zone cells in response to electric fields [70]. These data suggest that the sub-populations of cells in the meniscus are inherently different but these differences may be masked by the extracellular matrix in explant culture. Isolated cells lack natural cellular morphology and contact with native extracellular matrix components, whereas explants maintain the cells in the context of the extracellular matrix and associated signaling molecules. Important differences have been noted in the ability of cells to move through two-dimensional and three-dimensional culture systems, particularly due to the barriers presented by collagen networks [71]. In a recent study, fetal, juvenile and adult bovine meniscal cells showed similar proliferation rates and migration abilities in a monolayer micro-wound model. However, fetal and juvenile meniscal repair model explants showed improved repair strength over time while adult explants did not improve [44], further showing the capacity of these two model systems to reveal different information.

These model systems provide valuable information on the cellular response of the meniscus to inflammatory cytokines and growth factors, allowing a careful study of proliferation, migration and matrix deposition under well-controlled environmental conditions. These studies will help to inform future *in vivo *studies on mechanisms to promote meniscal repair. However, the direct translatability of these studies to *in vivo *applications is limited by the fact that the joint environment is more complicated, including the presence of many different cell and tissue types and a variety of inflammatory factors that are produced in the joint following meniscal injury. In addition, altered metabolism in all joint tissues and altered mechanical loading effects must be considered for successful *in vivo *studies.

There are few *in vivo *meniscal repair studies that have assessed cell migration and proliferation and extracellular matrix deposition. Several animal models of avascular meniscal tears have shown that either autologous or allogenic chondrocytes in a scaffold are necessary for the formation of reparative matrix tissue in the lesion and integration of cells into the native meniscus [72-74]. Animals treated with scaffolds alone resulted in increased cellularity of fibroblast-like cells at the edges of the lesion but no repair tissue in the interface [74]. Adipose-derived mesenchymal stem cells (ASCs) placed in rabbit avascular meniscal lesions prior to suturing, increased the healing rate and yielded an increase in the cellularity of meniscal fibrochondrocytes in the repair tissue [75]. Alternatively, several *in vivo *studies have demonstrated the need for a vascular supply to promote healing of meniscal lesions, inducing proliferation of vessels, endothelial cells and mesenchymal cells, and resulting in fibrovascular scar tissue repair [13,76]. However, proliferation of endothelial cells by vascular endothelial growth factor (VEGF) coated sutures was not sufficient to promote healing of meniscal lesions in the avascular region of sheep menisci [77]. The importance of meniscal cell migration and proliferation in meniscal healing is evidenced by a study showing that donor cells from fresh meniscal allografts in the goat do not survive but the host cells migrate into the allograft and repopulate the transplant [78]. In addition, in canine menisci containing devitalized meniscal plugs, cells migrate across the bridging tissue and into the interface, ultimately migrating into the devitalized plugs, remodeling the matrix, and filling the interface with a hyaline-fibrocartilage matrix [29].

Cell migration and/or proliferation are necessary for endogenous meniscal healing and repair. In order to repair a meniscal tear, cells must repopulate the wound and synthesize new extracellular matrix to achieve integrative repair. However, if cells are not able to fill in the gap, as in the presence of inflammatory cytokines, synthesis of reparative tissue and integrative repair cannot occur. Additionally, IL-1 treatment up-regulates MMP activity that promotes the catabolism of the meniscal extracellular matrix [24,26,27,34]. Therefore, a variety of strategies, including blocking proinflammatory cytokines [25], inhibiting MMP activity [26,79], and/or using anabolic growth factors [24] to increase matrix synthesis and promote cellular proliferation, may be required to promote meniscal healing following an injury and to increase the success of tissue engineering constructs.

## Conclusions

In conclusion, we have shown that the inflammatory cytokines IL-1 and TNF-α suppress the proliferation of meniscal cells and suppress integrative meniscal repair, while TGF-β1 overall does not alter the proliferation of cells or meniscal repair. In inner zone meniscal cells, migration is increased by IL-1 treatment but not enough to overcome the suppression of proliferation and fill the micro-wound. However, all other factors did not alter cellular migration independent of proliferation. Therefore, the suppression of cellular proliferation by IL-1 and TNF-α may prevent integrative repair of meniscal lesions by decreasing cell accumulation in the wound, and consequently diminishing the available cell population that can mediate the synthesis of reparative tissue. Therefore, strategies that promote the proliferation of meniscal cells may be able to enhance integrative repair following injury and promote the success of tissue engineered constructs.

## Abbreviations

ANOVA: analysis of variance; ASCs: adipose-derived mesenchymal stem cells; BSA: bovine serum albumin; DMEM: Dulbecco's Modified Eagle's Medium; EdU: 5-ethynyl-2'-deoxyuridine; ELF: electroforce; FBS: fetal bovine serum; HEPES: 4-(2-hydroxyethyl)-1-piperazineethanesulfonic acid; HUVEC: human umbilical vein endothelial cells; IL-1: interleukin-1; MMP: matrix metalloproteinase; NBT: nitroblue tetrazolium chloride; NO: nitric oxide; OA: osteoarthritis; PBS: phosphate buffered saline; PLLA: poly-L-lactide; S-GAG: sulfated glycosaminoglycan; TE: tris ethylenediaminetetraacetic acid; TGF-β1: transforming growth factor-beta 1; TNF-α: tumor necrosis factor-alpha; VEGF: vascular endothelial growth factor.

## Competing interests

The authors declare that they have no competing interests.

## Authors' contributions

KMR participated in the design of the study, performed the micro-wounding and explant studies, and drafted the manuscript. NER performed the micro-wounding studies, histology, data analysis and statistical analysis, and helped draft the manuscript. REW wrote the Matlab code and helped with data analysis and manuscript preparation. JBW helped conceive of the study and draft the manuscript. FG helped conceive of the study, participated in the study design and coordination, and helped draft the manuscript. ALM helped conceive of the study, participated in the study design and coordination, performed the micro-wounding experiments, data and statistical analyses, and drafted the manuscript. All authors have read and approved the final manuscript.
